# Flow Diverter Treatment of Ruptured Basilar Artery Perforator Aneurysms: A Multicenter Experience

**DOI:** 10.1007/s00062-021-01133-y

**Published:** 2022-01-20

**Authors:** Samer Elsheikh, Markus Möhlenbruch, Fatih Seker, Ansgar Berlis, Christoph Maurer, Naci Kocer, Ala Jamous, Daniel Behme, Christian Taschner, Horst Urbach, Stephan Meckel

**Affiliations:** 1 Department of Neuroradiology, Medical Center, University of Freiburg, Breisacherstr. 64, 79106 Freiburg, Germany; 2 Department of Neuroradiology, Heidelberg University Hospital, Heidelberg, Germany; 3 Diagnostic and Interventional Neuroradiology, University Hospital Augsburg, Augsburg, Germany; 4 Department of Neuroradiology, Cerrahpasa Medical Faculty, Istanbul University-Cerrahpasa Istanbul, Istanbul, Turkey; 5 Institute of Neuroradiology, University Medical Center Göttingen, Göttingen, Germany; 6 University Clinic for Neuroradiology, University Hospital Magdeburg, Magdeburg, Germany; 7 Institut für diagnostische und Interventionelle Neuroradiologie, RKH Klinikum Ludwigsburg, Ludwigsburg, Germany

**Keywords:** Subarachnoid hemorrhage, Angiogram-negative subarachnoid bleeding, Perimesencephalic subarachnoid bleeding

## Abstract

**Purpose:**

Ruptured basilar artery perforator aneurysms (BAPAs) represent a very rare cause of subarachnoid hemorrhage and an under-reported subtype of cerebral aneurysm. There is no consensus for the optimal treatment strategy (conservative vs. surgical vs. various endovascular approaches). We aim to present a multicenter experience of BAPA treatment using flow-diverter (FD) stents.

**Methods:**

At five tertiary neurovascular centers, all cases of ruptured BAPAs treated by FD were retrospectively collected. Baseline imaging and clinical characteristics, complications, as well as early and long-term angiographic and clinical outcome (mRS) were analyzed.

**Results:**

Eighteen patients (mean age, 57 years; SD, ±10.7 years) with acute SAH related to a BAPA were treated using 18 FD stents. Aneurysms were detected on initial imaging study in 28%; delayed diagnosis was triggered by clinical deterioration due to rebleeding in 15%. No rebleeding after FD was seen, 28% developed FD-related ischemic complications. At long term (*n* = 16), overall mortality was 13% (2/16), and favorable outcome (mRS 0–2) was 81% (13/16). All BAPAs (*n* = 13) were completely occluded at long-term angiographic follow-up.

**Conclusion:**

In our multicenter experience, FD treatment of ruptured BAPAs appears to have comparable safety and efficacy outcomes to FD treatment of other ruptured posterior circulation aneurysms as well as to the conservative management of BAPAs. This treatment strategy for a ruptured BAPA achieved a high rate of angiographic occlusion and favorable clinical outcome; however, as the conservative management also seems to offer similar clinical outcomes an individualized treatment decision is warranted. Future prospective studies comparing both approaches are required.

**Supplementary Information:**

The online version of this article (10.1007/s00062-021-01133-y) contains supplementary material, which is available to authorized users.

## Introduction

Perforating artery aneurysms of the anterior circulation are a rare subtype of intracranial aneurysms (prevalence < 1%) [1, 2]. Basilar artery perforator aneurysms (BAPAs) are even less common, with a recent systematic literature review by Granja et al. [1] describing only 56 cases of non-trunk type BAPAs since their initial description in 1996 [3]. Categorizing BAPAs is a challenging task due to a potential overlap with so-called blister-like aneurysms of the basilar artery trunk. Aboukais et al. described BAPAs as those whose neck is entirely located on a perforator artery without direct involvement of the basilar trunk [4]. Recently, Satti et al. proposed a comprehensive classification system based on the angiographic description of four distinctive types of BAPAs, which also included small blister-like aneurysms arising from the basilar trunk adjacent to a perforating artery branch but not involving the origin of a perforating artery (type I) [2].

BAPAs are a rare cause of subarachnoid hemorrhage (SAH). Their diagnosis requires a high degree of suspicion as they are often easily missed due to their small size (85% ≤ 3 mm), partial thrombosis and small caliber feeding vessels on initial CTA, MRA, and even DSA studies [1, 2, 5]. Given the rarity of these aneurysms and their natural history, an ideal approach to treatment remains poorly defined. Various treatment options have been reported in case reports and small series, including microsurgical clipping, endovascular embolization using coils or liquid embolic agents, and flow diversion (FD) within the basilar artery, each with its own unique challenges and risks. Conservative treatment is an additional management option and multiple prior reports have suggested a high rate of spontaneous resolution of BAPAs [6–9]; however, the rehemorrhage rate of BAPAs is reported in the order of 10–15%, which may result in death or severe disability [5, 6, 9, 10].

In this study, we present the multicenter experience derived from five large tertiary care neurovascular centers in the treatment of acutely ruptured BAPAs using FD stents.

## Materials and Methods

We performed a retrospective review of treated BAPAs in five tertiary care, neurointerventional university centers in two countries. Patients were included when treated with a FD for a ruptured BAPA (according to the classification by Satti et al. [2]) between 2013 and 2020. Data on patient demographics, clinical and radiological presentation, procedural data and postprocedural course of each patient were analyzed. Angiographic outcome after FD treatment was analyzed according to the O’Kelly-Marotta grading scale [11]. We also collected clinical outcome and follow-up data at least 90 days following treatment. Due to the retrospective nature of this study, the diagnostic and therapeutic management of included patients was not standardized. The diagnostic work-up of all patients included a digital subtraction angiography (DSA) including a rotational DSA and 3D-reconstructions of the intracranial vessels, which have contact to the SAH or show any suspicious findings. The treatment of each patient was performed according to the local standards of each center and individual operators. Patient consent was obtained in retrospect, when possible, at most centers. At one center informed consent for patient data publication was obtained as part of a local prospective registry.

## Results

### Patients

We identified 18 patients (8 females), who were treated using FD stents between 2013 and 2020 for ruptured BAPAs. Patient demographics and baseline characteristics are summarized in Table 1. A detailed description of individual patients is provided in Online Resource 1.

**Table 1:** Basic patient and aneurysm data

Demographics	Mean (range, SD) or % (*n*/*N*)
Age, years	57 (40–78 ±10.7)
Female	44.4% (8/18)
Hunt and Hess grade 1–2	72.2% (13/18)
Hunt and Hess grade 3–4	27.8% (5/18)
Fisher grade 1–2	22.2% (4/18)
Fisher grade 3–4	77.8% (14/18)
Rebleeding prior to diagnosis	22.2% (4/18)
Aneurysm identified on initial examination	27.8% (5/18; CTA, 2; CTA and DSA, 1; DSA, 2)
Aneurysm identified on repeat examination	72.2% (13/18; CTA, 2; DSA, 6; MRI, 1; unknown, 4)
Aneurysm size (mm)	1.7 (0.7–5)
Classification according to Satti et al. [2]	I: 16.7% (3/18), IIa: 5.6% (1/18), IIb: 50.0% (9/18), III: 27.8% (5/18)

### Presentation, Early Clinical Course and Diagnosis of Basilar Artery Perforator Aneurysm

All patients presented with a thunderclap headache due to an acute SAH. In two patients, repeat thunderclap headache was reported, suggestive of a rebleeding prior to hospitalization and one patient also presented with a Terson syndrome. Most patients showed severe SAH patterns on the initial imaging study (78%, Fisher grade 3–4), and only 17% presented with a perimesencephalic bleeding pattern.

A DSA was performed in all cases on presentation as part of the acute management of SAH. The perforator aneurysm was diagnosed in 28% of patients in the initial imaging study, whereas in 72% of cases the initial DSA did not reveal any aneurysm. In these cases, the aneurysm was discovered on repeat CT-angiography and/or DSA (median 14 days, range: 4–66 days) following the initial presentation. A retrospective re-evaluation of the initial DSA confirmed that all negative results were true negative ones.

In 15% (2/13) of patients with a delayed diagnosis, the repeat examination was triggered by a clinical deterioration due to a rebleeding. In the remaining patients (11/13) the repeat imaging study was performed as a routine follow-up examination before discharge. On initial discovery, the majority of the aneurysms were tiny (mean size 1.7 mm). The majority of aneurysms (50%) incorporated the perforating artery in the dome (Satti type IIb) or the aneurysm was located as a fusiform lesion along the course of the perforating artery (Satti type III, 28%).

Thus, the total number of cases suffering from a rebleeding prior to treatment was 22% (4/18), 2 cases prior to hospitalization and the initial vascular imaging. In these two patients, the aneurysm was discovered in the initial examination. In additional 2 patients, the aneurysm was discovered following the negative initial examination.

### Flow-diverter Treatment and Antiplatelet Medication

All patients received antiplatelet therapy that was initiated either before or during the procedure. The exact regimen varied according to the treating center and the time interval between diagnosis and treatment of the BAPA (for detailed individual regimens see Online Resource 2).

28% (5/18) patients received antiplatelet drugs before the procedure (range: 1–5 days): aspirin (100 mg) and clopidogrel (75 mg) were used in 2 patients (#4, #17), prasugrel 40 mg in 2 patients (#15, #16) and a single loading bolus of aspirin 300 mg and clopidogrel 300 mg in 1 patient (#6). The decision for delayed treatment was based on an individualized benefit-risk assessment of the need for immediate treatment vs. the need for adequate loading with antiplatelet medication.

72% (13/18) patients were loaded with antiplatelet medication during the procedure: 9 (#8–15, #18) patients received a weight-adjusted bolus of tirofiban followed by a maintenance dose for 12 h and 8 patients (#1–3, #5, #7–10) received an intravenous bolus of aspirin (100 mg/500 mg) with or without administration of clopidogrel via nasogastric tube. In addition, 12 patients (#1–6, #8, #10–11, #15–17) received an intravenous bolus of heparin (range, 2000–5000 IU).

All procedures were performed with the patient under general anesthesia via a transfemoral approach. The choice of flow diverter was left to the discretion of the treating physician. The following types of FD stents were utilized: FRED (*n* = 6), FRED X (*n* = 3), p48 HPC (*n* = 1), Pipeline (*n* = 1), Pipeline Shield (*n* = 3), Silk Vista Baby (*n* = 2), Surpass Evolve Stent (*n* = 1), Surpass Neuroendograft (*n* = 1).

All FD stents were deployed successfully. There was no procedural complication during the FD treatment. The immediate angiographic results showed complete (O’Kelly-Marotta occlusion score grade D) or near complete occlusion (O’Kelly-Marotta occlusion score grade C) in 33% (6/18) of treated BAPAs (Table 2).

**Table 2:** Angiographic and Clinical Outcomes after FD Therapy

Outcome	% (*n*/*N*)
*Angiographic outcome*	
Immediate adequate occlusion (OKM C/D)	33.3 (6/18)
Long-term adequate occlusion (OKM C/D)	100 (13/13)^a^
*Clinical outcome (mRS)*	
Favorable outcome (mRS 0–2) at discharge	72.2 (13/18)
Favorable outcome (mRS 0–2) at long-term	81.2 (13/16)^b^
*Mortality*	12.5 (2/16)^b^

^a^Angiographic long-term outcome was available in 13/18 patients. Clinical long-term outcome was available in 16/18 patients

^b^*OKM* O’Kelly-Marotta occlusion score

All patients received postoperative long-term antiplatelet therapy: two patients (#1–2) who received low thrombogenicity coated FD stents (p48 HPC and Pipeline Flex Shield) received long-term aspirin monotherapy, another two patients (#15–16) received long-term prasugrel monotherapy and one patient (#12) received ticagrelor monotherapy. The remaining patients received dual antiplatelet therapy (aspirin 100 mg plus either clopidogrel 75 mg daily or ticagrelor 90 mg twice daily) for 6 months followed by aspirin 100 mg daily monotherapy for varying durations (12 months to life-long).

### Early Neurological and Non-neurological Clinical Course

During hospital stay 17% (3/18) patients developed vasospasm requiring intra-arterial therapy. They were all treated using balloon angioplasty.

28% (5/18) patients developed ischemic complications during the hospital stay. Four of these patients (#1, 7, 9–10) suffered from FD-related ischemia, while in two patients (#7, 12) SAH related delayed cerebral ischemia was noted. In all but one (#1) of these patients, a FD without low thrombogenicity coating was used. Further information including the clinical outcomes of all patients with ischemic complications are summarized in Online Resource 2 and 3.

33% (6/18) developed hydrocephalus requiring an external ventricle drainage. In all cases, the external ventricle drains were placed before commencing the antiplatelet treatment. Two required a later permanent cerebrospinal fluid shunting. Changes to the antiplatelet medication plan at the time of permanent shunting were not available. There were no repeated hemorrhagic events (aneurysm related or otherwise) following stenting. One patient (#5) suffered from a Terson syndrome related to SAH in the acute phase with subsequent reduced visual acuity.

In addition, the following non-neurological clinical events were noted: two patients developed severe pneumonia (#11–12); the latter of which (#12) developed an acute respiratory distress syndrome with fatal outcome.

The clinical status at discharge (Table 2) was favorable in 72% (13/18; mRS 0, *n* = 5; mRS 1, *n* = 4; mRS 2, *n* = 4). Five patients had an unfavorable outcome (mRS 4, *n* = 1; mRS 5, *n* = 2), and two patients died during hospital stay (mRS 6).

### Clinical Course After Discharge

Following discharge one patient (#3) who had received a coated FD stent (PED Flex Shield device) suffered from a delayed pontine ischemia under aspirin monotherapy (100 mg daily) and dabigatran (110 mg twice daily, given for atrial fibrillation) on day 43 following the treatment (Online Resource 2–3). This patient subsequently had complete recovery (long-term mRS 1) after off-label intravenous thrombolysis (72 mg rTPA). Another patient (#7; mentioned above) required permanent cerebrospinal fluid shunting 70 days posttreatment. Another patient suffered from a dysfunction of the cerebrospinal fluid shunt with the need for subsequent operative revision of the shunt (#11).

### Clinical and Angiographic Long-term Outcome

**Fig. 1:**
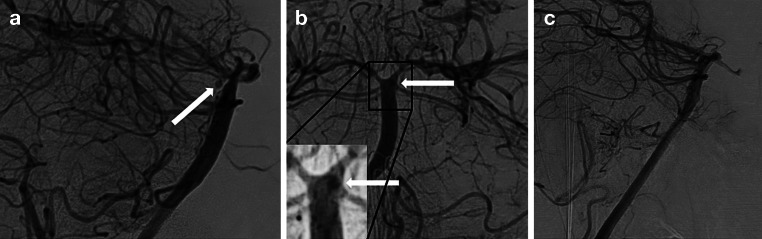
DSA of the basilar artery lateral view (**a**) showing a small circular BAPA (*arrow*) located on the dorsal aspect of the basilar artery (patient #2). DSA, ap view (**b**), with a high contrast zoomed insert showing the aneurysm (*arrows*) projected behind the basilar artery. 6‑month follow-up DSA lateral view of the same patient showing complete occlusion (O’Kelly-Marotta occlusion score D) of the BAPA

**Fig. 2:**
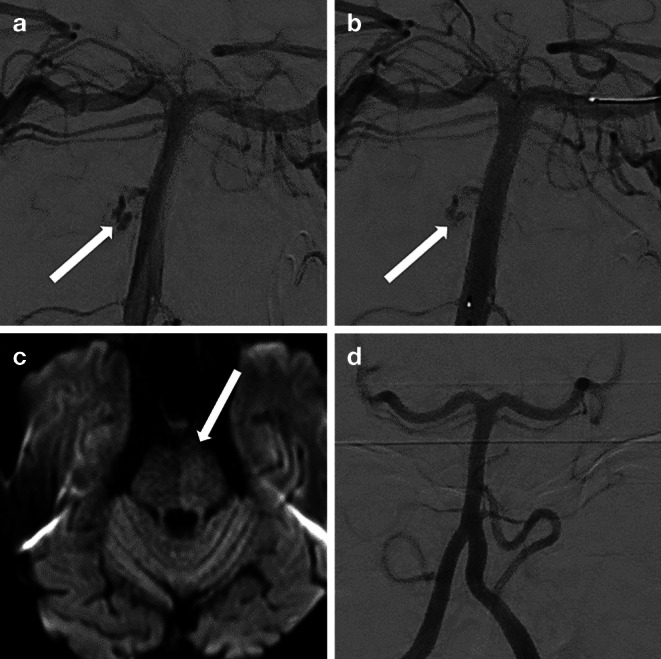
DSA of the basilar artery ap view (**a**) showing a possible active bleeding from a ruptured BAPA (patient #3). The patient suffered from two episodes of clinical worsening in the 2 h before the DSA. DSA ap view following implantation of the FD (**b**) with reduction of the contrast extravasation. Axial DW MR image (**c**) showing an increased signal intensity in the left paramedian area of the pons due to acute perforator artery occlusion. 6‑month follow-up DSA (**d**) showing complete occlusion (O’Kelly-Marotta occlusion score D) of the BAPA

Clinical and angiographic long-term outcomes are summarized in Table 2. Exemplary cases in Fig. 1 and 2.

Clinical long-term follow-up was available in 16/18 patients for at least 90 days (median [IQR], 90 [90–414] days, range, 90–2751 days). The remaining two patients (#14, 18) were treated for less than 90 days at the time of manuscript writing. The patient with Terson syndrome during the acute phase suffered from continued reduced visual acuity (#5). Two patients suffered from residual hemiparesis (#1, #7). One patient suffered from an impairment of memory and concentration (#2). Favorable long-term functional outcome (mRS 0–2) was achieved in 81% (13/16).

A long-term follow-up DSA after a median of 180 (IQR, 90–203; range, 9–390 days) was available in 13/16 surviving patients. A complete aneurysm occlusion (OKM grade D) was observed in all available cases (Table 2). Three of the surviving patients (#6, 14, and 18), who were treated for less than 180 days at the time of writing the manuscript, did not yet receive the scheduled follow-up angiographic examination.

## Discussion

BAPA is a rarely reported cause of acute SAH. In this study, we aimed to pool a multicenter experience in the treatment of these aneurysms using FD stents. In our cohort, 18 patients were treated for a ruptured BAPA, which comprises to our knowledge the largest published case series of FD treatment for this type of aneurysm. There were no intraprocedural complications. Before discharge, four patients (Online Resource 3) suffered from FD-related ischemia. After discharge, another patient suffered from a delayed perforator ischemia on day 43 following treatment under single antiplatelet treatment. The overall long-term mortality rate was 13% (2/16).

BAPAs exhibit a small aneurysm size, small feeding vessels in cases of distal or non-trunk type, and the possibility of spontaneous thrombosis after rupture at the time of diagnosis. This makes them difficult to diagnose, which in turn may lead to an underreporting of these aneurysms as the cause of the acute SAH [12, 13]. In our series, we performed a rotational DSA with 3D-reconstructions of suspected vessel territories and a retrospective re-evaluation of the negative initial vascular imaging in all patients. The re-evaluation showed that all negative results in the initial DSA examinations were true negatives. Nonetheless, in our series, only 28% of the aneurysms were diagnosed on the initial angiographic imaging study, which is in line with recent systematic reviews of non-trunk BAPAs by Granja et al. [1] and of posterior circulation perforator aneurysms by Gardijan et al. [14] reporting diagnosis of the aneurysm on the first DSA study in 50% and 39%, respectively. In absence of a confirmed rebleeding, we tried to perform the repeat DSA after confirmation of resolution of SAH-related vasospasm, using transcranial Doppler. The decreased flow and image quality during vasospasm may potentially lead to a false negative result.

Early rebleeding of ruptured BAPAs prior to the diagnosis in the initial or repeat DSA was relatively common in 22% of our series (two patients prior to hospitalization and two patients following the initial negative vascular imaging). This rate is at the upper range compared to rebleeding rates reported in aneurysmal SAH patients in general (8–23%) [15, 16]. The latter may be in part attributed to the initial nondiagnosis and subsequent rehemorrhage that was triggering the diagnosis and eventually treatment of the ruptured BAPA. In a recent literature review of conservative treatment of BAPA a low rate of rebleeding was reported (approximately 10%). The difference is probably due to a possible underdiagnosis of these aneurysms, publication bias and/or due to the low number of cases in each collective.

The exact regimen for antiplatelet therapy with FD treatment in SAH was not standardized in our series. In most cases, a pretreatment loading with single or dual antiplatelet agents, alternatively a glycoprotein IIb/IIIa inhibitor was administered, followed by 6 months of dual antiplatelet therapy and then long-term monotherapy. 22% did not receive dual antiplatelet therapy. In our cohort, this heterogeneity did not seem to affect complications or outcomes following FD treatment of BAPAs. The heterogeneity of treatment regimens and its lack of effect on the clinical outcomes were similarly reported in a recent meta-analysis of FD stent treatment of acutely ruptured aneurysms [17]. A concern about using antiplatelet therapy in the acute phase is the potentially increased risk for hemorrhagic complications. This is of particular concern in cases requiring further operative procedures in the acute phase (e.g. for cerebrospinal fluid drainage). Seven% (16/223) hemorrhagic events following FD treatment of ruptured aneurysms were reported in the above-referenced meta-analysis [17], more than half of these hemorrhages (11/223) were not aneurysm related. Thus, the timing of external ventricular drainage should preferably precede the endovascular treatment. This seems to minimize hemorrhagic events associated with external ventricular drainage [18]. An alternative strategy maybe the use of thrombogenicity-reduced coated FD stents such as the PED Flex Shield [19] or the p48 hpc device [20] combined with antiplatelet monotherapy in acutely ruptured aneurysms. In our series, no rebleeding events were observed following antiplatelet therapy and as well as no in-stent thrombosis. This could be attributed to the application of antiplatelet therapy in standard dose and may be explained by the low number of included patients. In spite of an immediate angiographic incomplete occlusion, a direct protective effect of FD was observed in our cohort. Gardijan et al. [14] reported a similar effect.

FD-related ischemic events in the acute phase were encountered in 28% of our cohort. This rate is in the order of other published reports on the use of FD stents for ruptured posterior circulation aneurysms. In a recent meta-analysis, the total complication rate for posterior circulation ruptured aneurysms treated with flow diverter was 27% [17]. In another systematic review of non-saccular posterior circulation aneurysms treated by FD, the overall rate of periprocedural ischemia was 23% [21]. Likewise, high rates of ischemic complications were also encountered in other reports of ruptured perforator aneurysms of the posterior circulation treated conservatively (32%), or by endovascular therapy using FD stents and mostly stent-in-stent techniques (20%) [14]. The overall high strokes rates are most likely related to the large number of perforators in the posterior circulation. Covering these perforators with a FD stent may cause short-term or long-term ischemic complications due to perforator occlusion.

Our cohort showed an excellent occlusion rate on long-term follow-up angiography (100%) and favorable clinical outcome (mRS 0–2) in 81%. Similarly high occlusion rates for BAPAs (94%) were reported following endovascular treatment by the use of single stents (10%), telescoping stents (stent-in-stent, *n* = 45%) or FD stents (45%) in a recent review of the literature comprising only small retrospective case series (1–5 cases each) [14]. Overall, favorable outcome with these different stent/FD treatment strategies was 90% in their review.

The natural history of BAPAs remains unclear. Conservative treatment seems to be a valid alternative, as spontaneous resolution of these aneurysms has been described in many smaller series [1, 7, 14, 22]. Despite lower occlusion rates (70%) compared to endovascular treatment using stents/FD (94%), clinical outcome showed no statistically significant difference with favorable outcome of 77% (conservative) vs. 90% (endovascular therapy) in a recent systematic review of small case series [14]. In this review, ischemia was often observed with conservative treatment (32%); however, 56% of these were associated with a failed endovascular treatment attempt. Rebleeding may occur with a conservative approach (10%), and mortality was 6%. In view of a lack of evidence for a clear treatment decision between a conservative approach (watch and wait strategy) and endovascular FD stent treatment, future clinical research with larger sample sizes is definitely required. Such a study should ideally be designed as a randomized trial or a least as a prospective multicenter registry, which may be a difficult undertaking given that BAPA is such a rare disease and cause of SAH.

Our study was limited by the retrospective nature, small sample size and the heterogeneity of the treatment protocols that were left to the individual operator’s discretion. Moreover, angiographic and clinical outcomes were self-adjudicated by the contributing centers.

## Conclusion

In our multicenter experience, FD treatment of ruptured BAPAs appears to have comparable safety and efficacy outcomes to FD treatment of other ruptured posterior circulation aneurysms as well as to the conservative management of BAPAs. This treatment strategy for a ruptured BAPA achieved a high rate of angiographic occlusion and favorable clinical outcome; however, as the conservative management also seems to offer similar clinical outcomes an individualized treatment decision is warranted. Future prospective studies comparing both approaches are required.

## Supplementary Information

Supplementary Table 1: Individual Clinical and Angiographic Presentation of Patients

Supplementary Table 2: Individual Data from Treatment and Follow-up

Supplementary Table 3: Causes and Outcomes of Ischemic Complications (acute and delayed)
