# Supplementary material for: Flow Diverter Treatment of Ruptured Basilar Artery Perforator Aneurysms: A Multicenter Experience
Source: Clin Neuroradiol. 2022 Jan 20;32(3):783–9. doi: 10.1007/s00062-021-01133-y (PMC9424161; doi:10.1007/s00062-021-01133-y)
Supplement: Supplementary file 1 — Supplementary Table 1: Individual Clinical and Angiographic Presentation of Patients [file 62_2021_1133_MOESM1_ESM.pdf]

Samer Elsheikh [1], Markus Möhlenbruch [2], Fatih Seker [2], Ansgar Berlis [3], Christoph Maurer [3], Naci Kocer [4], Ala Jamous [5], Daniel Behme [5,6], Christian Taschner [1], Horst Urbach [1], Stephan Meckel [7,1]

Author Affiliations:

1. Department of Neuroradiology, Medical Center – University of Freiburg, Faculty of Medicine, University of Freiburg, Germany.
2. Department of Neuroradiology, Heidelberg University Hospital, Germany.
3. Diagnostic and Interventional Neuroradiology, University Hospital Augsburg, Germany.
4. Department of Neuroradiology, Cerrahpasa Medical Faculty, Istanbul University-Cerrahpasa Istanbul, Turkey.
5. Institute of Neuroradiology, University Medical Center Goettingen, Goettingen, Germany.
6. University Clinic for Neuroradiology, Universtiy Hospital Magdeburg, Magdeburg Germany.
7. Institut für diagnostische und Interventionelle Neuroradiologie. RKH Klinikum Ludwigsburg, Ludwigsburg Germany

Corresponding Author:
Samer Elsheikh, M.D.
E-mail: samer.elsheikh@uniklinik-freiburg.de

Supplementary Table 1: *Individual Clinical and Angiographic Presentation of Patients*

Pat. #	age	Hunt and Hess Grade	Fisher Grade	Perimesencephalic pattern	Satti classification	Initial Diagnosis	Re-bleeding
1	70-79	1	2	yes	IIb	no	Yes
2	40-49	2	3	no	III	yes / CTA	No
3	70-79	1	3	yes	III	no	Yes
4	50-59	3	3	no	IIa	no	No
5	40-49	2	4	no	I	yes / CTA and DSA	No
6	60-69	2	3	no	IIb	no	No
7	60-69	2	3	no	III	yes / DSA	Yes
8	60-69	1	4	no	I	no	No
9	70-79	3	4	no	III	yes / DSA	Yes
10	50-59	4	4	no	IIb	no	No
11	60-69	4	4	no	III	no	No
12	50-59	3	4	no	IIb	yes / CTA	No
13	50-59	1	3	no	IIb	no	No
14	40-49	2	3	no	IIb	no	No
15	40-49	2	2	no	IIb	no	No
16	40-49	1	1	yes	IIb	no	No
17	40-49	1	2	no	IIb	no	No
18	50-59	2	3	no	I	no	No
