# Supplementary material for: Flow Diverter Treatment of Ruptured Basilar Artery Perforator Aneurysms: A Multicenter Experience
Source: Clin Neuroradiol. 2022 Jan 20;32(3):783–9. doi: 10.1007/s00062-021-01133-y (PMC9424161; doi:10.1007/s00062-021-01133-y)
Supplement: Supplementary file 2 — Supplementary Table 2: Individual Data from Treatment and Follow-up [file 62_2021_1133_MOESM2_ESM.pdf]

Samer Elsheikh [1], Markus Möhlenbruch [2], Fatih Seker [2], Ansgar Berlis [3], Christoph Maurer [3], Naci Kocer [4], Ala Jamous [5], Daniel Behme [5,6], Christian Taschner [1], Horst Urbach [1], Stephan Meckel [7,1]

Author Affiliations:

1. Department of Neuroradiology, Medical Center – University of Freiburg, Faculty of Medicine, University of Freiburg, Germany.
2. Department of Neuroradiology, Heidelberg University Hospital, Germany.
3. Diagnostic and Interventional Neuroradiology, University Hospital Augsburg, Germany.
4. Department of Neuroradiology, Cerrahpasa Medical Faculty, Istanbul University-Cerrahpasa Istanbul, Turkey.
5. Institute of Neuroradiology, University Medical Center Goettingen, Goettingen, Germany.
6. University Clinic for Neuroradiology, Universtiy Hospital Magdeburg, Magdeburg Germany.
7. Institut für diagnostische und Interventionelle Neuroradiologie. RKH Klinikum Ludwigsburg, Ludwigsburg Germany

Corresponding Author:
Samer Elsheikh, M.D.
E-mail: samer.elsheikh@uniklinik-freiburg.de

Supplementary Table 2: *Individual Data from Treatment and Follow-up*

Pat	Flow-Diverter		Medication (dose in IU or mg), duration			Complication (etiology or treatment)	Angiographic outcome (OKM Scale)		Clinical Outcome (mRS)	
#	Type	Size (mm)	pre-procedural	intra-procedural	post-procedural		intial	FU	at discharge	> 90d
1	p48 HPC	3.00x12	no	HEP (5000), ASS (500)	ASS (500, 5d; then 100, life)	Infarct (FD-rel.)	A2	D	4	1
2	Pipeline Shield	3.50x14	no	HEP (5000), ASS (500)	ASS (100), 6mth	VSM (EVT)	A3	D	2	2
3	Pipeline Shield	3.50x14	no	HEP (5000), ASS (500)	ASS (100), 6mth; CLO (75), 2y	delayed infarct (FD-rel.)	A3	D	0	1
4	Surpass Evolve	2.50x15	ASS (100), CLO (75)	HEP (5000)	ASS (100) 2 y, CLO (75), 6mth	no	A3	D	1	0
5	Surpass Neuroendograft	4.00x20	no	HEP (5000), ASS (500), CLO (600)	ASS (100), 18mth; CLO (75), 6mth	VSM (EVT)	B3	D	2	0
6	Pipeline Shield	2.50x14	ASS (300), CLO (300)	HEP (5000)	ASS (100) 18mth; CLO (75), 6mth	VSM (CON)	A3	NA	1	1
7	Pipeline	3.50x20	no	ASS (500)	ASS (100); CLO (75)	VSM (EVT), Infarct (FD/VSM-rel.). hydrocephal.	D	D	1	1
8	Silk Vista Baby	3.25x15	no	HEP (5000), ASS (100), TFN	ASS (100), TIC (2x90)	no	D	D	1	0
9	Silk Vista Baby	3.25x25	no	ASS (100), TFN	ASS (100); TIC (2x90)	Infarct (FD-rel.), hydrocephal.	B3	mRS 6	6	6
10	FRED	3.50x07	no	HEP (2000), ASS (500), TFN	ASS (100), life; CLO (75), 6mth	Infarct (FD-rel.)	A3	D	5	2

11	FRED	3.50x07	no	HEP (5000), TFN	ASS (100), life; CLO (75), 6mth;	pneumonia	A3	D	5	3
12	FRED	3.00x14	no	TFN	TIC (2x90)	VSM (CON), Infarct (VSM-rel.), pneumonia	C3	mRS 6	6	6
13	FRED X	3.50x07	no	TFN	ASS (100), life; TIC (2x90), 6mth	no	A3	D	2	1
14	FRED X	3.00x14	no	TFN	ASS (100), life; TIC (2x90), 6mth	no	A3	NA	2	NA
15	FRED	3.50x07	PRA (40)	HEP (5000), TFN	PRA, 2y	no	D	D	0	0
16	FRED	3.50x07	PRA (40)	HEP (5000)	PRA, 2y	no	D	D	0	0
17	FRED	3.50x07	ASS (100), CLO (75)	HEP (5000)	ASS (100), life; CLO (75), 6mth	no	D	D	0	0
18	FRED X	3.50x07	no	TFN	ASS (100), life; TIC (2x90), 6mth	no	A3	NA	0	NA

ASS = aspirin, CLO = clopidogrel, d = days, CON = conservative management, EVT = endovascular therapy, FD = Flow Diverter, HEP = heparin, life = life-long, mth = months, NA = not available, OKM = O'Kelly-Marotta occlusion score, PED = Pipeline embolization device, PRA = prasugrel, TFN = tirofiban given intravenously in weight-adapted scheme, TIC = ticagrelor, VSM=vasospasm, y = years
