# Supplementary material for: Flow Diverter Treatment of Ruptured Basilar Artery Perforator Aneurysms: A Multicenter Experience
Source: Clin Neuroradiol. 2022 Jan 20;32(3):783–9. doi: 10.1007/s00062-021-01133-y (PMC9424161; doi:10.1007/s00062-021-01133-y)
Supplement: Supplementary file 3 — Supplementary Table 3: Causes and Outcomes of Ischemic Complications (acute and delayed) [file 62_2021_1133_MOESM3_ESM.pdf]

Samer Elsheikh [1], Markus Möhlenbruch [2], Fatih Seker [2], Ansgar Berlis [3], Christoph Maurer [3], Naci Kocer [4], Ala Jamous [5], Daniel Behme [5,6], Christian Taschner [1], Horst Urbach [1], Stephan Meckel [7,1]

Author Affiliations:

1. Department of Neuroradiology, Medical Center – University of Freiburg, Faculty of Medicine, University of Freiburg, Germany.
2. Department of Neuroradiology, Heidelberg University Hospital, Germany.
3. Diagnostic and Interventional Neuroradiology, University Hospital Augsburg, Germany.
4. Department of Neuroradiology, Cerrahpasa Medical Faculty, Istanbul University-Cerrahpasa Istanbul, Turkey.
5. Institute of Neuroradiology, University Medical Center Goettingen, Goettingen, Germany.
6. University Clinic for Neuroradiology, Universtiy Hospital Magdeburg, Magdeburg Germany.
7. Institut für diagnostische und Interventionelle Neuroradiologie. RKH Klinikum Ludwigsburg, Ludwigsburg Germany.

Corresponding Author:
Samer Elsheikh, M.D.
E-mail: samer.elsheikh@uniklinik-freiburg.de

Supplementary Table 3*: Causes and Outcomes of Ischemic Complications (acute and delayed)*

Patient #	Cause of Ischemic complication	Stent Coating	mRS at Discharge	mRS > 90 days
1	FD-related	low thrombogenicity coated	4	1
3	FD-related (delayed on day 43)	low thrombogenicity coated	0	1
7	FD-related and Delayed cerebral ischemia	not coated	1	1
9	FD-related	not coated	6	6
10	FD-related	not coated	5	2
12	Delayed cerebral ischemia	not coated	6	6
